# Editorial: Spatio-temporal molecular mechanisms regulating synapse function and neural circuit dynamics

**DOI:** 10.3389/fnmol.2025.1564028

**Published:** 2025-02-07

**Authors:** Tomohisa Hosokawa, Yoshiyuki Kubota, Tetsuya Takano

**Affiliations:** ^1^Department of Pharmacology, Kyoto University Graduate School of Medicine, Kyoto, Japan; ^2^Section of Electron Microscopy, Supportive Center for Brain Research, National Institute for Physiological Sciences, Okazaki, Japan; ^3^The Graduate University for Advanced Studies (SOKENDAI), Okazaki, Japan; ^4^Support Unit for Electron Microscopy Techniques, Research Resources Division, RIKEN Center for Brain Science, Wako, Japan; ^5^Division of Molecular Systems for Brain Function, Institute for Advanced Study, Medical Institute of Bioregulation, Kyushu University, Fukuoka, Japan; ^6^PRESTO, Japan Science and Technology Agency, Saitama, Japan

**Keywords:** synapse, transcriptome analysis, proteomic, neuronal circuit, signaling/signaling pathways

Understanding how synaptic function and neuronal circuit dynamics are regulated is a cornerstone of neuroscience as these processes are pivotal for information transmission, memory formation, and adaptive responses to environmental changes. They offer insights into how the brain processes information, adapts to experiences, and responds to injuries, such as through mechanisms like synaptic plasticity in learning, neural regeneration after trauma, and adaptive circuit remodeling in response to environmental changes. These mechanisms are also central to understanding the pathophysiology of psychiatric and neurological disorders. While significant advances have been made such as the development of high-resolution imaging techniques and the identification of key molecular regulators, the precise regulation of synaptic properties and neural circuits across temporal and spatial dimensions remains insufficiently understood. Addressing these challenges is crucial for uncovering the molecular mechanisms underlying brain plasticity and advancing novel therapeutic approaches for neurological and psychiatric disorders.

This Research Topic focuses on the spatiotemporal molecular mechanisms that regulate synaptic function and neural circuit dynamics. It brings together a diverse range of studies aiming to bridge existing knowledge gaps. By delving into the molecular basis of synaptic properties and their dynamic changes, this Research Topic offers critical insights into synaptic function regulation and circuit plasticity, with the broader goal of advancing our understanding of brain plasticity and its implications for neurological disorders.

Recent advances in genetic analysis have uncovered the complexity of neural networks and the diversity of synaptic functions through detailed gene expression profiles in neurons. The review article titled “*Molecular diversity and functional dynamics in the central amygdala*” by Yeh et al. highlights the intricate cellular and molecular landscape of the central amygdala (CeA), a critical brain region involved in processing emotions and modulating behaviors related to fear and anxiety. The authors introduce distinct neuronal populations within the CeA and summarize recent advancements in transcriptomic technologies that reveal the functional diversity of these cells. These molecular profiles provide insights into the potential role of the CeA in emotional regulation and highlight avenues for therapeutic exploration in neuropsychiatric disorders. The review also suggests directions for future molecular studies to deepen our understanding of CeA dynamics.

In recent years, significant progress has also been made in understanding the complexity of neural circuits and synapses, driven by advancements in molecular techniques, proteomic profiling, and data-driven research. Recent advances in proteomic profiling have led to significant progress in understanding the complexity of neural circuits and synapses. The review article “*Synaptic proteomics decode novel molecular landscape in the brain*” by Ito et al. delves into cutting-edge proteomic techniques that are reshaping our understanding of synaptic biology. The authors highlight the use of mass spectrometry combined with spatial and proximity-based labeling methods such as BioID and APEX to map the complex protein composition within synapses. These methods provide high-resolution insights into the diverse and dynamic molecular landscape of synapses, revealing functional adaptations important for synaptic connectivity. The detailed exploration of these proteomic approaches underscores their potential in elucidating the molecular mechanisms underlying synaptic diversity and how they contribute to the overall function of neural circuits, providing a pathway to understanding complex brain function.

The review article “*Neuromodulator regulation and emotions: insights from the crosstalk of cell signaling*” by Tsuboi et al. explores how neuromodulatory mechanisms regulate synaptic activity and emotional behavior, extending the discussion of proteomic advances by focusing on how these molecular mechanisms translate into functional and behavioral outcomes. The authors provide an in-depth discussion on how neuromodulators such as dopamine and acetylcholine influence synaptic plasticity and behavioral outcomes by modulating key signaling pathways. Specifically, the review highlights the application of phosphorylation proteomics to study the pathways that govern the excitatory-inhibitory balance in neural circuits, thereby impacting emotional regulation. The insights gained from these studies underscore the molecular complexity of neuromodulator-driven changes in neural activity and their relevance to neuropsychiatric disorders. The review also suggests future directions in neurochemical analysis, emphasizing the need for deeper exploration of signaling crosstalk to fully understand the mechanisms underlying emotional regulation. Complementing these insights, the review article “*KANPHOS: Kinase-associated neural phospho-signaling database for data-driven research*” by Kannon et al. introduces KANPHOS, a web-based database designed to support research into neural phospho-signaling. KANPHOS consolidates data on protein kinases, their phosphorylated substrates, and disease associations, offering a valuable resource for studying protein phosphorylation in neural function. By integrating diverse datasets, including phospho-proteomics, mutant models, and disease associations, KANPHOS enables comprehensive pathway analyses. It also features an application programming interface (API) functionality for integration with external tools, facilitating a holistic approach to studying signaling mechanisms in neurological disorders. This review underscores the importance of advanced proteomic technologies, phosphorylation analysis, and data-driven tools in expanding our understanding of neural circuits and synaptic function. By integrating approaches from protein composition analysis to pathway regulation and database resources, these studies provide a comprehensive view of the molecular mechanisms governing neural circuits.

Recent advancements in molecular neuroscience have deepened our understanding of the mechanisms driving neurological disorders. The review article “*Morphogenetic theory of mental and cognitive disorders: the role of neurotrophic and guidance molecules”* by Primak et al. propose that deviations in brain morphogenesis during critical developmental windows underpin mental and cognitive disorders. Disruptions in processes like cellular differentiation, migration, and synaptic patterning are linked to structural and functional neural impairments, highlighting the importance of early intervention to mitigate neuropsychiatric risks. Complementing this perspective, the article “*Role of C9orf72 hexanucleotide repeat expansions in ALS/FTD pathogenesis”* by Geng and Cai explores the genetic basis of amyotrophic lateral sclerosis (ALS) and frontotemporal dementia (FTD). They identify hexanucleotide repeat expansions in the C9orf72 gene as a major cause of familial ALS and FTD, emphasizing how toxic RNA foci and dipeptide repeat proteins drive neurodegeneration, offering potential therapeutic targets. Further, the study “*Optogenetic elevation of postsynaptic cGMP in the hippocampal dentate gyrus enhances LTP and modifies mouse behaviors”* by Borovac et al. demonstrate that optogenetically increasing postsynaptic cGMP enhances long-term potentiation (LTP) and alters behaviors in mice. This underscores the therapeutic potential of modulating cGMP signaling for cognitive deficits. These findings suggest that modulating cGMP signaling pathways could be a potential therapeutic strategy for cognitive impairments associated with neurological conditions.

Collectively, the articles presented in this Research Topic collection offer a detailed theoretical framework for understanding the spatio-temporal regulation of synaptic function, including how molecular signaling pathways interact with genetic and developmental processes. These insights reveal critical mechanisms underlying brain plasticity, synaptic connectivity, and the dynamics of neural circuits, providing a foundation for future therapeutic strategies targeting neurological and psychiatric disorders. By integrating a wide range of methodologies—including proteomics, transcriptomics, phosphorylation signaling analysis, and optogenetics—these reviews elucidate the complex molecular mechanisms underlying brain plasticity, synaptic connectivity, and neural circuit dynamics. From examining synaptic proteins and neuromodulator signaling to exploring genetic and morphogenetic factors contributing to neurodegeneration and mental health disorders, these contributions underscore the necessity of a multidisciplinary approach. Such an integrated perspective is crucial for identifying the molecular determinants of brain function, advancing our understanding of neuropsychiatric disorders, and developing innovative therapeutic interventions targeting structural and functional anomalies in the brain.

